# Clean care is safer care: from a glonal challenge to a WHO patient safety programme

**DOI:** 10.1186/1753-6561-5-S6-P260

**Published:** 2011-06-29

**Authors:** M-N Chraïti, B Allegranzi, S Bagheri Nejad, C Kilpatrick, E Mathai, D Pittet

**Affiliations:** 1 Infection control programme and WHO Collaborating Center on Patient Safety, University of Geneva Hospitals, Geneva, Switzerland; 2 Clean Care is Safer Care, WHO Patient Safety, WHO Headquarter, Geneva, Switzerland

## Introduction / objectives

The First Global Patient Safety Challenge *Clean Care is Safer Care* (CCiSC) was launched in 2005 and is now a WHO Patient Safety Programme with the aim to prevent a frequent and major adverse event in care delivery?healthcare associated-infection (HCAI).

## Methods

Efforts have focused on hand hygiene improvement using a three-pronged approach: 1) raising awareness of HCAI among health professionals and solutions for its prevention; 2) securing political commitment at governmental level to make HCAI prevention a health priority; 3) developing a range of technical tools to support hand hygiene programmes at the facility level, according to the WHO validated multimodal strategy for hand hygiene improvement.

## Results

To date, 124 of 147 WHO member states have pledged their support to CCiSC to reduce HCAI. As an extension of the CCiSC work, the *SAVE LIVES: Clean Your Hands* annual global campaign was launched in 2009. In 2010, over 12,000 healthcare facilities worldwide signed up to the initiative. The accompanying technical toolkit has been widely used and adopted and often adapted to local conditions. In addition, 42 countries worldwide have implemented their own national hand hygiene campaign.

## Conclusion

Future plans are to scale up CCiSC to include other areas of infection control with surveillance as the next focus. But efforts will continue to promote actively the importance of the sustainability of hand hygiene improvement.

## Disclosure of interest

None declared.

